# Detection of Stimulus Displacements Across Saccades is Capacity-Limited and Biased in Favor of the Saccade Target

**DOI:** 10.3389/fnsys.2015.00161

**Published:** 2015-11-27

**Authors:** David E. Irwin, Maria M. Robinson

**Affiliations:** Department of Psychology, University of IllinoisChampaign, IL, USA

**Keywords:** perceptual stability, saccadic eye movements, object correspondence, memory, visual acuity

## Abstract

Retinal image displacements caused by saccadic eye movements are generally unnoticed. Recent theories have proposed that perceptual stability across saccades depends on a local evaluation process centered on the saccade target object rather than on remapping and evaluating the positions of all objects in a display. In three experiments, we examined whether objects other than the saccade target also influence perceptual stability by measuring displacement detection thresholds across saccades for saccade targets and a variable number of non-saccade objects. We found that the positions of multiple objects are maintained across saccades, but with variable precision, with the saccade target object having priority in the perception of displacement, most likely because it is the focus of attention before the saccade and resides near the fovea after the saccade. The perception of displacement of objects that are not the saccade target is affected by acuity limitations, attentional limitations, and limitations on memory capacity. Unlike previous studies that have found that a postsaccadic blank improves the detection of displacement direction across saccades, we found that postsaccadic blanking hurt the detection of displacement *per se* by increasing false alarms. Overall, our results are consistent with the hypothesis that visual working memory underlies the perception of stability across saccades.

## Introduction

Our eyes scan the world via rapid, saccadic eye movements several times each second in order to bring high-resolution foveal vision to bear on objects of interest in the visual world. Even though the retinal positions of objects in the world change dramatically with each saccade, we generally perceive a stable visual world, with objects maintaining a constant direction with respect to the viewer. How this quality of perception is achieved has been debated for over a century. Relatedly, given that the retinal positions of all objects change during a saccade, how is instability (i.e., actual change in an object’s spatial position during a saccade) detected?

Over the years many different theories have been proposed to account for these phenomena (for a review, see Bridgeman et al., 1994). Early theories proposed that a cancellation mechanism used information about eye position to compensate for the retinal image changes that accompany saccades, subtracting changes in eye position from retinal image shifts to remap object positions across saccades (e.g., Von Holst and Mittlestaedt, 1950; Sperry, 1950). A mismatch between oculomotor information and retinal image motion would produce a perception of instability, as happens when the eye is passively displaced by pressing on the canthus, for example (e.g., Helmholtz, 1867/1925).

Cancellation theories were called into question when it was discovered that information about eye position is not very accurate and is not well time-locked to the saccade (for a review, see Matin, 1986). For example, displacing a visual stimulus during a saccade is typically difficult to detect unless the displacement is large relative to the amplitude of the saccade; Bridgeman et al. (1975) found that stimulus displacements as large as 33% of saccade amplitude were frequently undetected. Furthermore, perceptual stability was found to rely heavily on the presence of visual landmarks in the environment and not just information about eye position (e.g., Matin, 1976; Bridgeman and Graziano, 1989; Hayhoe et al., 1991; Deubel et al., 1998), with visual information dominating oculomotor information when they conflicted. Visual landmarks provide reference information about the relative positions of objects with respect to each other (e.g., Gibson, 1950, 1966, 1979), and thus stability can be signaled by constancy in these relative positions and instability by change in relative object positions across the saccade (e.g., Haber, 1985).

Given these findings, in recent years several theories of perceptual stability (and instability) across saccades that rely on establishing object correspondence across saccades have been proposed as alternatives to classical cancellation theories (Tas et al., 2012). For example, McConkie and Currie (1996) proposed that the perception of stability across saccades relies largely on a local evaluation process centered on the saccade target object, rather than on a remapping and evaluation of all of the objects in the scene (see also Deubel et al., 1984, 1996, 1998; Deubel, 2004; Hollingworth et al., 2008; Tas et al., 2012). According to their *saccade target object theory*, viewers selectively encode features of the saccade target object prior to executing a saccade, and then after the saccade they attempt to locate this object within a limited retinal search region near the fovea. If the saccade target object can be found, then stability is assumed by the perceptual system (cf. MacKay, 1973). Displacing the target during the saccade reduces the likelihood that it will be found within the search region when the eyes land, leading the viewer to perceive instability. In support of this theory, Currie et al. (2000) found in a picture-viewing experiment that displacing an object near the saccade target was much more detectable than displacing everything in the picture except the saccade target. Similarly, Bridgeman (1981) and Brune and Lücking (1969) found that when a picture was shifted during a saccade, only the object near the saccade endpoint appeared to move, rather than the entire picture, suggesting that the evaluation of instability across saccades is largely confined to the saccade-landing region.

Further support for object correspondence theories of perceptual stability across saccades has been provided by studies showing that perception of saccade target displacement is improved if object correspondence across the saccade is broken, which has the effect of violating the assumption of object stability across the saccade and thereby facilitating displacement detection. As noted earlier, displacing a visual stimulus during a saccade is typically difficult to detect, but Deubel et al. (1996) found that presenting a blank screen during and after the saccade before re-presenting the target at its shifted position greatly improved detection of its displacement. Demeyer et al. (2010) and Tas et al. (2012) showed further that merely changing some characteristic of the saccade target object (such as its form or polarity) improved the detection of its displacement across a saccade. These manipulations all have the effect of breaking object correspondence across the saccade, thereby violating the assumption of object stability and facilitating displacement detection.

Although the results summarized above provide good support for the hypothesis that the perception of stability across saccades relies on a local evaluation process centered on the saccade target object, they do not conclusively demonstrate that the perception of stability across saccades depends only on the saccade target object and not on other objects in the visual world. For example, although the results of Currie et al. (2000) are consistent with the saccade target object theory, they also seem consistent with an account in which multiple objects are evaluated across a saccade at relatively low spatial resolution, with the saccade target object having priority in the perception of displacement. Such an account also predicts that displacements of the saccade target object should be most noticeable, while explaining detection of background element displacement in terms of low resolution or low priority evaluation of the entire scene.

This possibility was investigated by Irwin and Robinson (2014), who found evidence that the positions of multiple objects are indeed maintained and evaluated across saccades. In their Experiment 2, two or six uniquely-colored circles were presented on a display, preceded by a color preview cue at fixation that indicated which circle should be targeted by a saccade. This pre-saccadic array was erased when a saccade was detected and was then replaced by only a single circle, either the saccade target or one of the context objects chosen at random. The single circle was either presented in its original, pre-saccadic location, or displaced by 2°. Subjects had to respond whether the stimulus had been displaced or not. Irwin and Robinson (2014) found that displacement detection for the saccade target object was higher than displacement detection for the context objects, but displacement detection for the context objects was significantly greater than chance and did not differ across array sizes of two vs. six items. Participants did not know which object would be probed on each trial, so this finding suggests that the positions of all of the items in the display had been maintained across the saccade. Irwin and Robinson (2014) concluded that the positions of multiple objects are maintained across saccades and evaluated with respect to perceptual stability, with priority given to the saccade target because of visual acuity and attentional focus being better near the fovea.

The Irwin and Robinson (2014) study had several limitations. A relatively small number of observations were collected from each participant and only one displacement size (2°) was employed. As noted above, detection of stimulus displacement across saccades depends on the ratio between stimulus displacement and saccade amplitude, with the threshold for reliable displacement detection being typically 10–33% of saccade amplitude (Bridgeman et al., 1975). The participants in the Irwin and Robinson study executed 6–8° saccades, so the stimulus displacement they used was relatively high (25–33% of saccade amplitude). These limitations may have made it difficult to detect differences in performance. To address these limitations, in the present studies a relatively large number of observations were collected from each participant and several displacement distances were employed, with the goal of addressing with more sensitivity the questions of how many objects are maintained across saccades and with what degree of accuracy. In addition, we examined the effect of a factor that has been shown to improve displacement detection of the saccade target, namely postsaccadic target blanking, to determine whether its effect is limited to the saccade target or whether it improves detection of context element displacements as well.

## Experiment 1

### Method

#### Participants

Six students from the University of Illinois community were recruited for this experiment. The participants reported normal or corrected to normal vision and were naïve as to the purpose of the experiment. Each subject received payment for completing 5 thirty-minute sessions. The research was approved by the University of Illinois Institutional Review Board and each participant signed an informed consent form before each experimental session.

#### Apparatus and Procedure

Eye movements were recorded with an EyeLink 1000 Plus camera-based eyetracker (SR Research Ltd., Mississauga, Ontario, Canada) with temporal resolution of 1000 Hz, spatial resolution of <0.01° RMS, and pupil size resolution of 0.2% of pupil diameter. Stimuli were presented on a Benq 24-inch LED monitor (also purchased from SR Research) with resolution of 1920 × 1080 pixels and refresh rate of 144 Hz. According to SR Research, this monitor has <2 ms latencies at all resolutions and refresh rates tested. At a refresh rate of 144 Hz the monitor was refreshed approximately every 7 ms, while saccade durations in our experiments were approximately 30–40 ms in duration. Participants’ heads were stabilized with a chinrest, fixed at 69 cm from the computer monitor. At this viewing distance the display subtended 42° horizontally and 24° vertically. Participants adjusted the height of their chair so that their eyes were centered with respect to the display monitor. The stimuli were filled color circles. A Minolta CS-100 Chroma Meter (Minolta Camera Company, Japan) was used to measure their luminance and chromaticity using the CIE color coordinate system. The display background was white, with a luminance of 255 cd/m^2^. Manual responses were made with a Response Pixx button box interfaced with the eyetracking computer. The experiments were programmed using the Experiment Builder software sold by SR Research for use with their Eyelink system.

**Figure 1:**
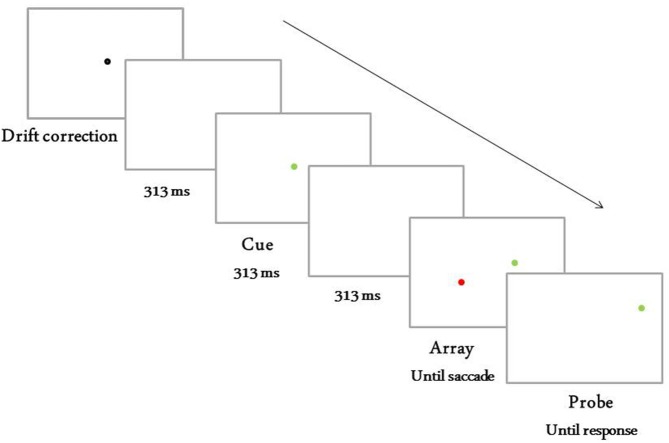
**Sequence of events for trials in Experiment 1.** Depicted is a target move trial with display size of two and movement in the same direction as the saccade.

Each experimental block of trials began with a nine-position calibration procedure in which the edges and center of the screen were fixated. Figure 1 depicts an example of the sequence of events on an experimental trial. Participants began each trial by pressing a button on the button box while fixating a drift correction dot that subtended 0.38° of visual angle. After the drift correction dot disappeared, a blank white screen was presented for 313 ms. Then a cue appeared at the center of the screen. The cue was a filled colored circle that subtended 0.5° of visual angle and that was assigned one of six colors, red (*x* = 0.619, *y* = 0.327, 53.3 cd/m^2^), blue (*x* = 0.150, *y* = 0.062, 28.8 cd/m^2^), black (5.5 cd/m^2^), cyan (*x* = 0.233, *y* = 0.340, 209 cd/m^2^), yellow (*x* = 0.386, *y* = 0.481, 241 cd/m^2^), or green (*x* = 0.317, *y* = 0.599, 154 cd/m^2^). The cue was presented for 313 ms and then a blank white screen was presented for 313 ms. Finally, an array consisting of two or six filled colored circles equal in size to the circle cue was presented. The color of each circle was pseudorandomly assigned to red, blue, black, cyan, yellow, or green, with the caveat that no two circles could be the same color and that one circle matched the color of the circle cue. Each circle was randomly assigned a position on one of two imaginary circles concentric about the central fixation and with radii of 4° or 6°. Potential circle locations corresponded to the 12 positions of a clock. Circles were never presented at the same clock position at different eccentricities.

Participants were informed that the circle in the array that shared the color of the circle cue was the saccade target, and they were instructed to make a saccade to the saccade target as soon as the array of circles appeared. An eye movement was classified online as a saccade when its velocity reached 50° per second; saccade detection triggered a display change, as described next.

During the saccade one of the four displacement conditions was initiated: either the saccade target remained on screen in its pre-saccadic location (target no move); the saccade target remained on screen but was displaced by some amount (target move); a context circle remained on screen in its pre-saccadic location (context no move); or a context circle remained on screen but was displaced by some amount (context move). This probe item remained visible until a response was given. Participants pressed one button on the response pad to indicate that they detected a displacement or a different button to indicate that they detected no displacement. On trials where the saccade target was probed the displacement distance was −3°, −2.25°, −1.5°, 0.75°, 0°, 0.75°, 1.5°, 2.25°, or 3° (negative values indicate that the displacement was in the opposite direction as the saccade while positive values indicate that the displacement was in the same direction as the saccade). On trials where a context circle was probed the displacement distance was −4°, −3°, −2°, −1°, 0°, 1°, 2°, 3°, or 4°. These displacement distances were determined through pilot testing. Positive and negative displacement distances were used to make the task more difficult by increasing the positional uncertainty of the probe item and also to produce a range of saccade landing distances from the probe, whose effect is analyzed below. Participants were not informed about the distribution of displacement distances, nor were they told that no displacement would occur on only 1/9 of the trials.

Participants completed five blocks of 72 trials each during each experimental session. Displacement condition (target probed vs. context probed), array size (2 vs. 6), and displacement distance (9 values) were counterbalanced but sequenced randomly within each block of trials.

### Results

Individual trial data were excluded from analysis if the subject did not follow instructions or if the experimental program failed to detect that a saccade had been made or updated the display too slowly. For example, trials were excluded if the saccade was not directed at the saccade target location (defined as a 30° wedge around the saccade target location); this occurred on 12.4% of trials. Trials were also excluded if the saccade amplitude was less than 1° (0.3% of trials) or for inappropriate button responses such as pressing the trial advance button rather than one of the response buttons (0.03% of trials). The display change was not always completed during the saccade to the target location, either because the software did not detect the saccade or because the time required to detect saccade onset and to update the display was longer than the saccade duration. In these cases the display change occurred within a fixation (i.e., during the post-saccadic fixation) rather than across fixations so these data were also excluded from analysis (0.9% of trials). This left 86.4% of the trials available for analysis.

Information about saccade latencies, durations, and amplitudes is presented in Table 1 for the two target distances in all three experiments. Target distance was manipulated merely to increase positional uncertainty and the number of possible display locations so it was not considered further in the remaining analyses.

**Table 1:** **Latency, duration, and amplitude of saccades directed at targets located 4° and 6° from fixation in experiments 1–3**.

		Latency 4°	Duration 4°	Amplitude 4°	Latency 6°	Duration 6°	Amplitude 6°
Experiment 1	Mean	188	32	3.4	195	39	5.1
	SEM	2.70	1.32	0.09	2.58	1.36	0.14
Experiment 2	Mean	152	31	3.4	161	38	5.0
	SEM	3.77	1.62	0.15	5.68	1.55	0.23
Experiment 3	Mean	197	32	3.7	207	38	5.2
	SEM	10.65	0.79	0.16	17.69	1.37	0.25

**Figure 2:**
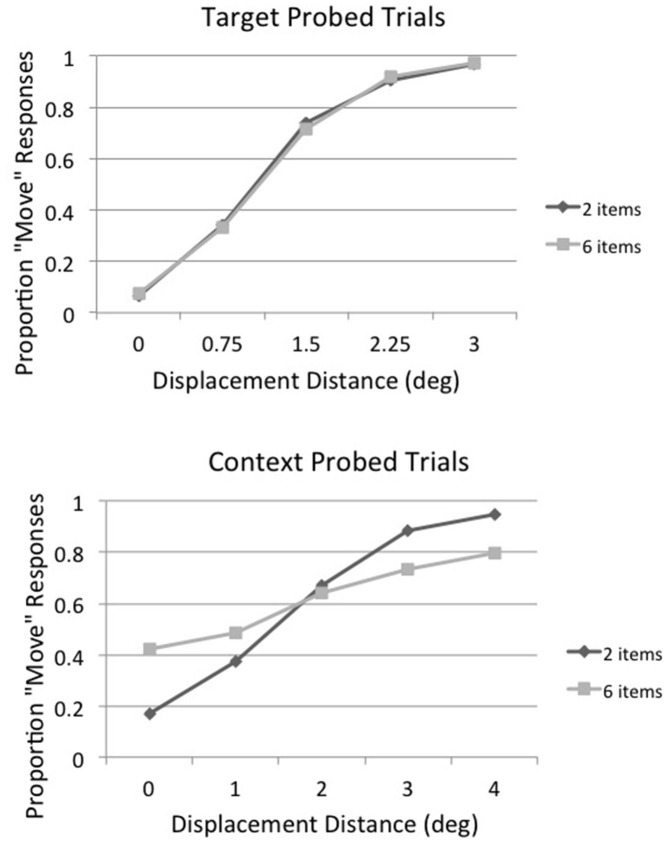
**Experiment 1: proportion of displacement (“move”) responses for target probed (top panel) and context probed (bottom panel) trials as a function of displacement distance and array size (2 vs. 6 items) averaged across subjects**.

Irwin and Robinson (2014) found that displacement direction *per se* did not influence displacement detection, so positive and negative displacement distances were averaged together in the main analysis to increase the number of observations per condition. The proportion of displacement (“move”) responses for target probed (top panel) and context probed (bottom panel) trials as a function of displacement distance and array size (2 vs. 6 items) averaged across subjects is shown in Figure 2. Inspection of Figure 2 shows that array size had essentially no effect on displacement detection when the saccade target object was probed but a very large effect when a context item was probed. There was a large increase in the number of false alarms (i.e., percentage of “move” responses on no move (0°) displacement trials) as array size increased on context probed trials and perhaps a difference in the upper asymptote as well. This implies that subjects had less precise information on whether the stimulus had been displaced or not on context probed trials when the array size was 6 rather than 2 and thus they may have engaged in more guessing behavior.

**Figure 3:**
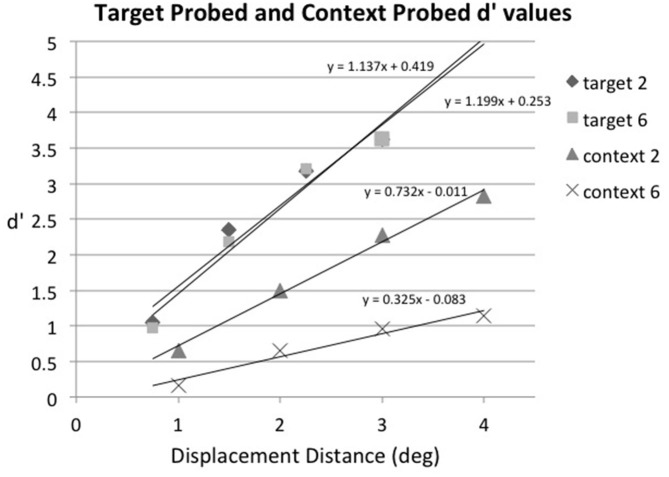
**Experiment 1: sensitivity (d′) as a function of displacement distance in degrees for target probed, array size 2 trials (target 2), target probed, array size 6 trials (target 6), context probed, array size 2 trials (context 2), and context probed, array size 6 trials (context 6), averaged across subjects.** The best-fitting straight line for each condition is also plotted.

Our initial analysis plan had been to find the threshold (75% detection point) for each condition, but given the large differences in false alarm rates across conditions this did not seem viable. So, instead, the displacement percentages were converted to d′ for further analysis, using the 0° displacement detection result for each condition as its false alarm rate. Note that because the no-displacement (0°) conditions and the displacement conditions were intermixed within each block of trials, the displacement conditions share a common no-displacement condition and thus a common false alarm rate (Kanwisher et al., 1996; Salthouse and Mitchell, 1989; Agus et al., 2010; Pratte et al., 2010). The results of this analysis are shown in Figure 3, along with the best-fitting straight line for each condition (i.e., target probed, array size 2; target probed, array size 6; context probed, array size 2; context probed, array size 6). The formulas for the best-fitting straight lines were used to calculate a threshold-like value for each condition; we chose a d′ of 1.35 for this purpose because it corresponds to a 75% level of accuracy when there is no bias (i.e., a hit rate of 75% and a correct rejection rate of 75%). We will call this value the threshold for purposes of convenience. The displacement distance that corresponded to a d′ of 1.35 for target probed, array size 2 trials was 0.79°; the corresponding displacement distances for the other conditions were 0.89° for target probed, array size 6 trials; 1.88° for context probed, array size 2 trials; and 4.71° for context probed, array size 6 trials.

For statistical purposes best-fitting straight lines were fit to the displacement condition data for each individual subject and then these were used to calculate the threshold displacement distance (i.e., the displacement distance that gave rise to a d′ of 1.35) for each experimental condition. The individual linear fits were very good, with *r*^2^ ranging from 0.75–1.0 across conditions (the mean *r*^2^ averaged across subjects and conditions was 0.93, *SD* = 0.07). In some cases the hit rate was 100% or the false alarm rate was 0%, so the individual subject data were corrected before d′ was calculated by adding 0.5 to all cells in the signal-detection matrix, regardless of content (Goodman, 1970), which Brown and White (2005) found is the optimal correction procedure for extreme discriminability measures. A two-way repeated-measures ANOVA was then conducted on the d′ threshold values with factors of displacement condition (target probed vs. context probed) and array size (2 vs. 6) and then Scheffe *post hoc* comparisons were made to examine contrasts of interest. Displacement thresholds were lower on target probed trials than on context probed trials, *F*_(1,5)_ = 74.18, *p* < 0.001, *MSE* = 0.486. In addition, displacement thresholds were lower when there were two items in the array than when there were six, *F*_(1,5)_ = 19.01, *p* < 0.01, *MSE* = 0.680. The interaction between displacement condition and array size was also significant, *F*_(1,5)_ = 13.17, *p* < 0.02, *MSE* = 0.846. The error term from the interaction was used to construct a Scheffe 95% confidence interval for comparing two means; this value was ± 1.06. Based on this, the displacement distance that corresponded to a d′ of 1.35 for target probed, array size 2 trials (0.79°) did not differ from that of target probed, array size 6 trials (0.89°), but it was significantly smaller than that for context probed, array size 2 trials (1.88°), which was significantly smaller than that for context probed, array size 6 trials (4.71°), thus verifying what is visually apparent in Figure 3.

**Figure 4:**
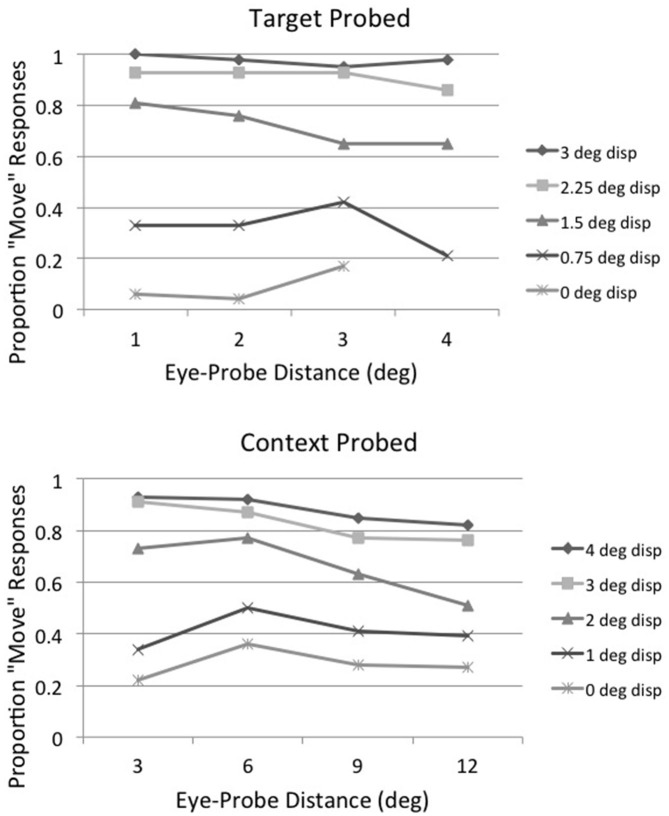
**Experiment 1: detection likelihood as a function of the eyes’ distance from the probed item for each displacement distance for target probed (top panel) and context probed (bottom panel) trials, averaged across subjects and array sizes**.

Although Irwin and Robinson (2014) found that displacement direction *per se* does not influence displacement detection, displacement direction does have an effect on the distance between the saccade endpoint and the position of the probe item, and this does influence displacement detection. In the present experiment, percent detection on displacement trials (i.e., excluding no-move trials) when the saccade target was probed was 77.8% when the probe moved in the direction opposite to the saccade and 68.9% when the probe moved in the same direction as the saccade. The pattern was reversed for context probed trials, with detection at 61.8% when the probe moved in the direction opposite to the saccade and 77.3% when the probe moved in the same direction as the saccade. Displacement direction had different effects on target probed and context probed trials because moving the probe in the direction opposite to the saccade on target probed trials moved it closer to the landing point of the saccade (1.65° vs. 2.65°) while moving the probe in the direction opposite to the saccade on context probed trials moved it farther away from the landing point of the saccade (8.42° vs. 5.56°). Thus, the distance between the saccade landing point (i.e., the position of the fovea) and the position of the probe item, rather than displacement direction *per se*, affects detection performance. This is illustrated in more detail in Figure 4, which shows that while the probability of detection increases as displacement distance increases, it decreases at each displacement distance as the distance between the saccade landing point and the position of the probe increases (except at the extremes, where detection is either at ceiling or below chance).

### Discussion

The results of Experiment 1 show that displacement of the saccade target object is detected more accurately than displacement of other objects in a display, and it is unaffected by the number of other objects in the display. This illustrates that the saccade target object has privileged status in detecting instability across saccadic eye movements, as hypothesized by others (e.g., Deubel et al., 1984, 1996, 1998; McConkie and Currie, 1996; Currie et al., 2000; Deubel, 2004; Irwin and Robinson, 2014). The displacement detection threshold for the saccade target object (0.8–0.9°) was within the range of other studies of displacement detection across saccades (approximately 17% of saccade amplitude in our case). Our results suggest that the positions of other elements in the display are also maintained and evaluated across a saccade, but with lower resolution. When only two items were present in the display and the context item was probed (instead of the saccade target), the displacement detection threshold was 1.88°, more than double the threshold when the saccade target was probed. This may have been due to the fact that the probe was farther away from the fovea when the context item was probed (7° on average) than when the saccade target was probed (2.15° on average). Objects farther from the fovea are seen with lower spatial resolution (e.g., Loschky et al., 2005; Strasburger et al., 2011), and thus there is more imprecision in people’s estimates of their locations (Rosenholtz et al., 2012). This would make it more difficult for participants to judge whether the context item had moved or not during the saccade. In addition, however, there seems to be a limit on the number of items that can be maintained and evaluated across a saccade, because displacement detection of context items was considerably worse when six items were in the display (threshold = 4.71°) rather than just two (threshold = 1.88°). The average distance between the saccade landing point and the position of the probe was constant across array sizes, so this difference cannot be attributed to acuity factors. Rather, it suggests that participants are unable to encode and remember the positions of six array items across the saccade. This is inconsistent with the conclusions of Irwin and Robinson (2014), who found no differences in accuracy as a function of array size in their experiment. They used only a single, large, displacement distance and had relatively few trials per experimental condition, however, so they may have lacked the power to detect a difference. The present results do support their conclusion that multiple objects are maintained and evaluated across saccades, but the accuracy with which this occurs is affected by the number of objects in the display. The fact that displacement detection of context items declined as the number of items in the visual field increased indicates that there is an attentional or memory capacity limitation on their influence in perceiving stability across saccades.

## Experiment 2

As described above, the threshold for detecting stimulus displacement across a saccade is typically quite high, requiring displacements of approximately 10–33% of the amplitude of the saccade in order to be detected reliably (for a review, see Bridgeman et al., 1994). However, Deubel and colleagues (Deubel and Schneider, 1994; Deubel et al., 1996, 1998) found that the threshold for detecting the direction of stimulus displacements was reduced substantially if a blank (empty screen) interval of 50–300 ms separated saccade onset and presentation of the postsaccadic stimulus. They hypothesized that precise information about the presaccadic target position and high-fidelity information about eye position are always available after a saccade, but are overridden if visual information is present immediately after the saccade. If the display is blank, then the perceptual system is able to use eye position information to update the spatial location of the saccade target, thereby improving displacement detection. In Experiment 2, we investigated whether inserting a blank period between saccade onset and the presentation of the post-saccadic probe would affect displacement detection across a saccade in the paradigm used in Experiment 1. Of particular interest was whether detection of context element displacement, as well as saccade target displacement, might improve because of access to previously ignored eye position information. Furthermore, because the results of Experiment 1 showed that displacement detection on context probed trials was affected by the number of items in the display, we used array sizes of 2 and 4 (rather than 2 and 6) to obtain additional information about capacity limits on performance.

### Method

#### Participants

The same six students who took part in Experiment 1 participated in this experiment as well. Each received payment for completing 7 thirty-minute sessions.

#### Stimuli and Procedure

The stimuli and procedure were identical to Experiment 1 except the displacement distances were changed to −2.25°, −1.5°, −0.75°, 0°, 0.75°, 1.5°, or 2.25° on trials when the saccade target was probed and to −4°, −3°, −2°, 0°, 2°, 3° or 4° on trials when a context item was probed. Participants were not informed about the distribution of displacement distances, nor were they told that no displacement would occur on only 1/7 of the trials. The presaccadic arrays contained either two or four items. On half of the target and context probe trials the display was erased when a saccade was detected and was kept blank for 300 ms before the postsaccadic probe was presented, while on the other half the probe was presented as soon as possible after saccade onset, as in Experiment 1. Displacement condition (target probed vs. context probed), blank condition (no-blank vs. blank), array size (2 vs. 4), and displacement distance (7 values) were counterbalanced but sequenced randomly within each block of trials. Participants completed four blocks of 112 trials each during each experimental session.

### Results

Individual trial data were excluded from analysis if the saccade was not directed at the saccade target location (21.4% of trials), if the saccade amplitude was less than 1° (0.7% of trials), or if the display change was not completed during the saccade to the target location on no-blank trials (1.6% of trials). This left 76.3% of the trials available for analysis.

**Figure 5:**
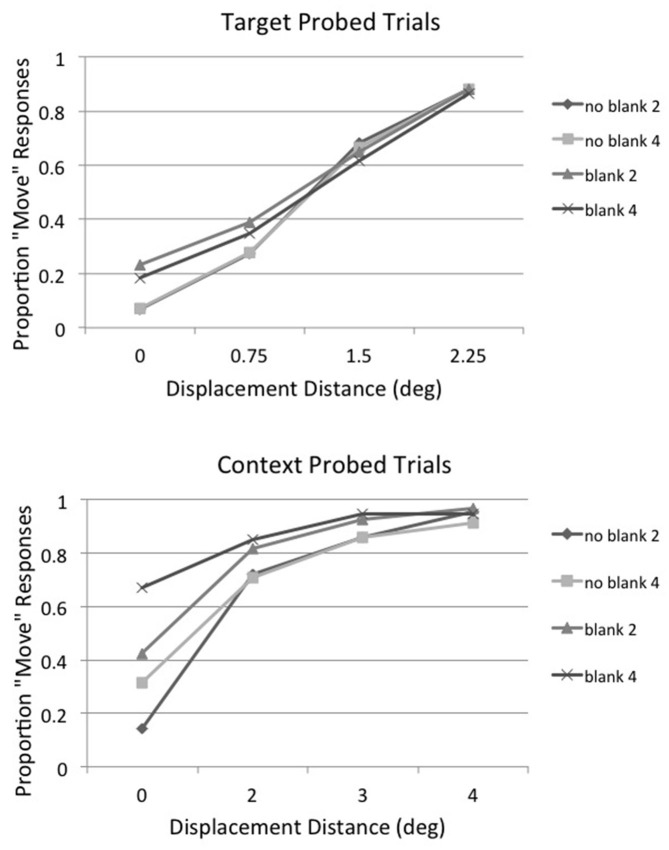
**Experiment 2: proportion of displacement (“move”) responses for target probed (top panel) and context probed (bottom panel) trials as a function of displacement distance for no blank, array size 2 trials (no blank 2), no blank, array size 4 trials (no blank 4), blank, array size 2 trials (blank 2) and blank, array size 4 trials (blank 4), averaged across subjects**.

**Figure 6:**
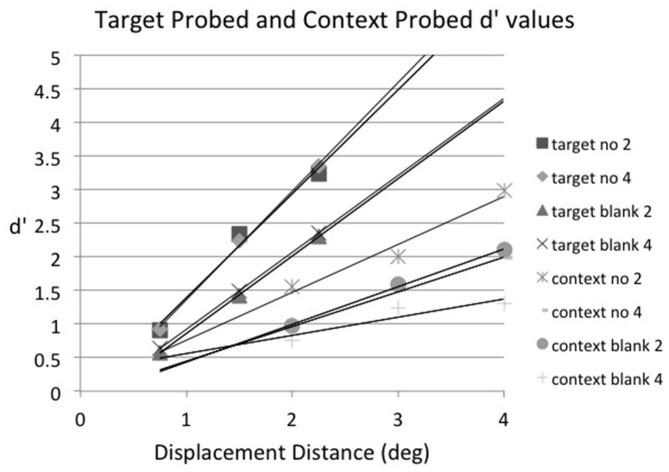
**Experiment 2: sensitivity (d′) as a function of displacement distance in degrees for target probed, no blank, array size 2 trials (target no 2), target probed, no blank, array size 4 trials (target no 4), target probed, blank, array size 2 trials (target blank 2), target probed, blank, array size 4 trials (target blank 4), context probed, no blank, array size 2 trials (context no 2), context probed, no blank, array size 4 trials (context no 4), context probed, blank, array size 2 trials (context blank 2), and context probed, blank, array size 4 trials (context blank 4), averaged across subjects.** The best-fitting straight line for each condition is also plotted.

As in Experiment 1, positive and negative displacement distances were averaged together in the main analysis to increase the number of observations per condition. The proportion of displacement (“move”) responses for target probed (top panel) and context probed (bottom panel) trials as a function of displacement distance, blank condition (no-blank vs. blank), and array size (2 vs. 4 items), averaged across subjects, is shown in Figure 5. Inspection of Figure 5 suggests that the postsaccadic blank increased the proportion of detection responses on context probed trials, but at the expense of increasing substantially the false alarm rate (i.e., responding “move” when no displacement occurred). Given the large differences in false alarm rate across conditions, the displacement percentages were converted to d′ for further analysis as in Experiment 1, using the 0° displacement detection result for each condition as its false alarm rate. These results are shown in Figure 6, along with the best-fitting straight line for each condition. To reduce clutter the regression formulas are not shown, but they were used calculate the displacement distance that produced the threshold value of d′= 1.35 in each condition, as in Experiment 1. The threshold displacement distances for target probed, no-blank trials was essentially the same regardless of array size (array size 2 trials = 1.0°; array size 4 trials = 0.99°). So, as in Experiment 1, array size had no effect on the displacement threshold when the saccade target was probed. This was also true when a blank was presented before the postsaccadic probe was presented (array size 2 trials = 1.51°; array size 4 trials = 1.42°). In contrast, the displacement threshold was higher when there were more elements in the display when a context item was probed instead of the saccade target (for no-blank trials, array size 2 = 1.82°; array size 4 = 2.85°; for blank trials, array size 2 = 2.64°; array size 4 = 4.26°).

For statistical purposes best-fitting straight lines were fit to the displacement condition data for each individual subject and then these were used to calculate the threshold displacement distance (i.e., the displacement distance that gave rise to a d′ of 1.35) for each experimental condition. The individual linear fits were generally very good (except for one subject in the context probed, array size 6 with blank condition), with *r*^2^ ranging from 0.35–1.0 across conditions (the mean *r*^2^ averaged across subjects and conditions was 0.94, *SD* = 0.12). As in Experiment 1, individual subject data were corrected by adding 0.5 to all cells in the signal-detection matrix to correct for hit rates of 100% and false alarm rates of 0% before d′ was calculated. A three-way repeated-measures ANOVA was conducted on the d′ threshold values with factors of displacement condition (target probed vs. context probed), blank condition (no blank vs. blank) and array size (2 vs. 4) and then Scheffe *post hoc* comparisons were made to examine contrasts of interest. The main effect of displacement condition was significant, *F*_(1,5)_ = 47.12, *p* = 0.001, *MSE* = 0.703, as displacement thresholds were lower on target probed trials than on context probed trials. The main effect of blank condition was also significant, *F*_(1,5)_ = 65.23, *p* < 0.001, *MSE* = 0.116, as displacement thresholds were lower on no-blank trials than on blank trials. The interaction between displacement condition and blank condition was significant, *F*_(1,5)_ = 7.32, *p* < 0.05, *MSE* = 0.161, because blanking caused thresholds to increase more on context probed trials than on target probed trials (Scheffe 95% confidence interval for the interaction comparison = 0.65 ± 0.60). The interaction between displacement condition and array size, *F*_(1,5)_ = 7.75, *p* < 0.05, *MSE* = 0.734 was also significant, because array size had no effect on thresholds when the saccade target was probed but caused thresholds to increase on context probed trials (Scheffe 95% confidence interval for the interaction comparison = 1.38 ± 1.27). Finally, the three-way interaction of displacement condition, blank condition, and array size, *F*_(1,5)_ = 8.82, *p* < 0.05, *MSE* = 0.039 was also significant, due to the fact that the blanking effect was larger on context probed array size 4 trials than in the other conditions (Scheffe 95% confidence interval for the interaction comparison = 0.83 ± 0.34).

### Discussion

The no-blank trials of Experiment 2 duplicated and extended the results of Experiment 1 in several ways. Displacement of the saccade target object was detected more accurately than displacement of other objects in the display and detection of these displacements was unaffected by the number of other objects in the display. This provides further evidence that the saccade target object has privileged status in detecting instability across saccadic eye movements. In contrast, detecting displacements of objects that were not targeted by the saccade (i.e., context items) was less accurate and was affected by the number of items in the display. When only two items were present in the display and the context item was probed (instead of the saccade target), the displacement detection threshold was 1.82°, almost double the threshold when the saccade target was probed (1°). These thresholds were very similar to those found in the first experiment (1.88° and 0.79° respectively). When four items were present in the display and a context item was probed the displacement detection threshold rose to 2.85°. This is lower than the threshold found in Experiment 1 for trials in which six items were present in the display (4.71°), providing further evidence that there is an attentional or memory capacity limitation on the influence of context items in perceiving stability across saccades.

The major purpose of Experiment 2 was to investigate whether adding a post-saccadic blank interval before presenting the probe might improve performance, as Deubel and colleagues found in their experiments (Deubel et al., 1996, 1998; see also Demeyer et al., 2010 and Tas et al., 2012). We found instead that the presentation of a post-saccadic blank hurt displacement performance, largely by increasing false alarms. This was true both when the saccade target was probed and when a context item was probed, and was most detrimental to performance when the array contained four objects and a context item was probed. The procedure that we used is different in several ways from the one used by Deubel et al. (1996, 1998), Demeyer et al. (2010) and Tas et al. (2012), however, and we believe that these differences are responsible for the different pattern of results across the studies. In our procedure, participants had to judge whether a stimulus moved or not. More than one stimulus was presented, the stimuli were arranged across virtual concentric circles surrounding fixation (thus requiring saccades of varying angular eccentricities), and sometimes the saccade target was probed while other times a context item was probed instead. In contrast, in the experiments of Deubel et al. (1996, 1998), Demeyer et al. (2010) and Tas et al. (2012), there was only one stimulus (the saccade target), all eye movements were horizontal, the saccade target *always* moved during the saccade, and subjects had to judge whether it moved to the left or to the right. The results of Deubel et al. (1996, 1998), Demeyer et al. (2010) and Tas et al. (2012) show clearly that inserting a post-saccadic blank improves people’s ability to judge which way the saccade target moved, whereas our results demonstrate that inserting a post-saccadic blank leads people to believe that a stimulus moved even when it did not move (as shown by increases in the false alarm rate). Why?

A series of papers by Deubel et al. (1998, 2010); Deubel (2004) may provide the answer. Deubel et al. (1998) found that the presence of a continuously visible landmark could override the beneficial effects of blanking and cause a stable saccade target appear to jump when it was blanked. Similarly, participants in Deubel et al. (2010) exhibited a strong bias to perceive movement of a blanked object when two objects were presented near the saccade target and one of them was blanked during the saccade, even if the non-blanked object moved and the blanked object did not. In our experiments, it seems possible that the display screen acted as a stable reference landmark, so after subjects completed their saccade and waited for the probe to appear on blank trials, they may have perceived the probe to be displaced even when it was not. A problem for this explanation is that Deubel (2004) found that a visual landmark had to be spatially near the saccade target to have any effect. The viewing conditions that we used were quite different from those used by Deubel, however, with Deubel using a background luminance of 2.2 cd/m^2^ and stimulus luminances of 25 cd/m^2^ whereas our background luminance was 255 cd/m^2^; the black frame of our display monitor had a luminance of 11 cd/m^2^, so the borders of our display may have provided a salient visual landmark. Another possibility is that participants in our study may have relied on their memory of the layout of items in the pre-saccadic display as a type of landmark. In our experiment, unlike the Deubel experiment, there was uncertainty about which item would be probed, so participants presumably tried to maintain a memory representation of the pre-saccadic array before the post-saccadic probe was presented. This memory representation could serve as a kind of landmark, thereby creating perceived displacement of the probed item following the post-saccadic blank. Of course, it is also possible that some other procedural difference might have contributed to the conflicting pattern of results across experiments; this requires further study.

In any case, our results do not seem consistent with the hypothesis that precise information about the presaccadic target position and high-fidelity information about eye position can be used after a saccade to update the spatial location of the saccade target (nor other objects in a display) when visual information is not immediately available after the saccade. If this hypothesis were correct, it seems that we should have found that subjects would be more accurate at judging whether the stimulus moved or did not move during the saccade because precise information about its pre-saccadic location would be available. Given that all of the studies that have found beneficial effects of blanking on spatial discrimination across saccades have used a motion direction discrimination task, it seems possible that a post-saccadic blank may aid discrimination of motion direction across saccades rather than improving knowledge of spatial position *per se*.

## Experiment 3

Experiment 3 was designed to address two possible concerns regarding the procedures that were used in the first two experiments. One concern is that there were unequal numbers of move and no-move trials. In Experiment 1 the stimulus was displaced on 89% of the trials and was not displaced on 11% of the trials, and in Experiment 2 these values were 86% and 14% respectively. It is possible that having so few no-move trials might have biased subjects to respond “move” on a disproportionate number of trials, producing a large number of false alarms, especially on context probed trials. To address this concern, in Experiment 3 we used equal numbers of move and no-move trials. A second possible concern about the procedures used in the first two experiments is that the saccade target was probed on 50% of the trials and a context item was probed on 50% of the trials. This meant that any given context item was probed less often than the saccade target (for array sizes larger than 2, anyway) and this may have led subjects to attend to the saccade target more than the context items, producing a benefit for detecting saccade target displacements over context item displacements. To address this concern, in Experiment 3 the saccade target was probed no more often than any given context item. A single set size of four items was used, with the saccade target being probed on 25% of the trials and a context item being probed on 75% of the trials.

### Method

#### Participants

Six new students were recruited to participate in this experiment. None had participated in either of the first two experiments and they were naïve with respect to the experimental hypotheses. Each received payment for completing 7 thirty-minute sessions.

#### Stimuli and Procedure

The stimuli and procedure were identical to Experiment 2 except that only one array size of four items was used, each item in the array had an equal probability of being probed (i.e., the saccade target was probed on 25% of the trials and a context item was probed on 75% of the trials), and no displacement occurred on half of the trials. As in the first two experiments, participants were not informed about the distribution of displacement distances or probe locations, nor were they told that no displacement would occur on 1/2 of the trials. As in Experiment 2, on half of the target and context probe trials the display was erased when a saccade was detected and was kept blank for 300 ms before the postsaccadic probe was presented, while on the other half the probe was presented as soon as possible after saccade onset. Displacement condition (target probed vs. context probed), blank condition (no blank vs. blank), and displacement distance (with half of the trials being no-displacement trials) were sequenced randomly within each block of trials. Participants completed five blocks of 96 trials each during each experimental session.

### Results

Individual trial data were excluded from analysis if the saccade was not directed at the saccade target location (19.2% of trials), if the saccade amplitude was less than 1° (0.7% of trials), or if the display change was not completed during the saccade to the target location on no-blank trials (1.6% of trials). This left 78.5% of the trials available for analysis.

**Figure 7:**
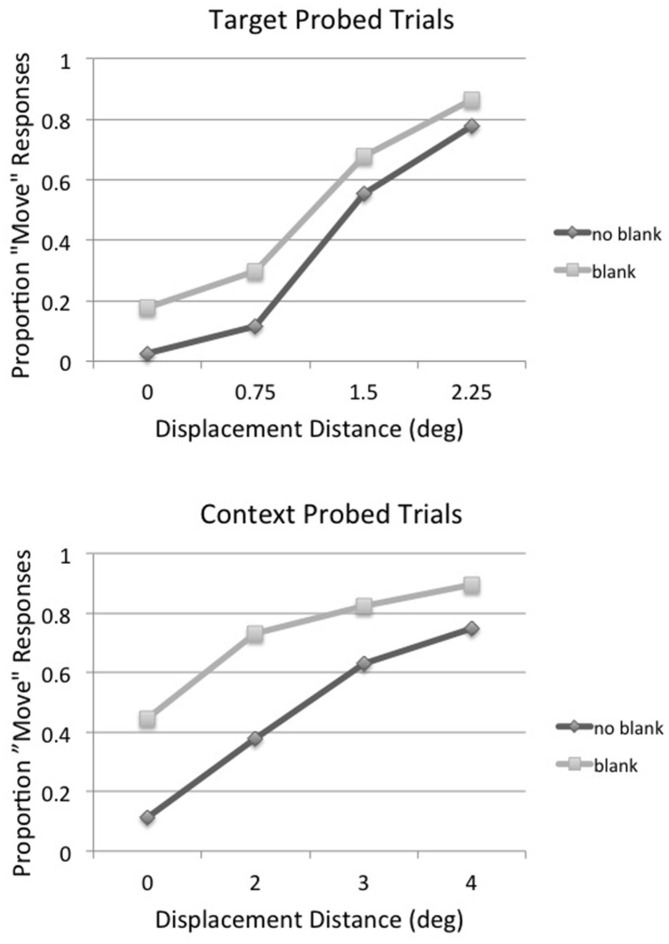
**Experiment 3: proportion of displacement (“move”) responses for target probed (top panel) and context probed (bottom panel) trials as a function of displacement distance for no blank and blank trials, averaged across subjects**.

**Figure 8:**
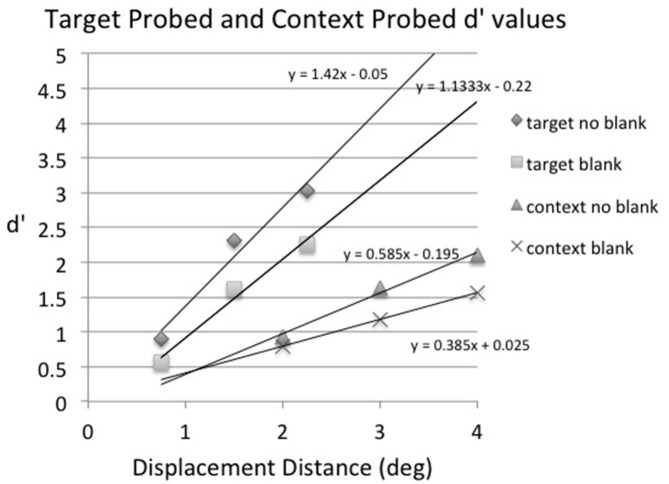
**Experiment 3: sensitivity (d′) as a function of displacement distance in degrees for target probed, no blank trials (target no blank), target probed, blank trials (target blank), context probed, no blank 2 trials (context no blank), and context probed, blank trials (context blank), averaged across subjects**.

As in the first two experiments, positive and negative displacement distances were averaged together in the main analysis to increase the number of observations per condition. The proportion of displacement (“move”) responses for target probed (top panel) and context probed (bottom panel) trials as a function of displacement distance and blank condition (no-blank vs. blank), averaged across subjects, is shown in Figure 7. The results were very similar to those of Experiment 2: the postsaccadic blank increased the proportion of detection responses, but at the expense of increasing substantially the false alarm rate (i.e., responding “move” when no displacement occurred). Given the large differences in false alarm rate across conditions, the displacement percentages were converted to d′ for further analysis as in Experiments 1 and 2, using the 0° displacement detection result for each condition as its false alarm rate. These results are shown in Figure 8, along with the best-fitting straight line for each condition. As in the first two experiments these were used to calculate the displacement distance that produced the threshold value of d′= 1.35 in each condition. The threshold displacement distances were very similar to those found for array size 4 trials in Experiment 2: the threshold for target probed, no-blank trials was 1.03° (compared to 0.99° in Experiment 2); the threshold for target probed, blank trials was 1.48° (compared to 1.42° in Experiment 2); the threshold for context probed, no-blank trials was 2.79° (compared to 2.85° in Experiment 2); and the threshold for context probed, blank trials was 3.74° (compared to 4.26° in Experiment 2).

For statistical purposes best-fitting straight lines were fit to the displacement condition data for each individual subject and then these were used to calculate the threshold displacement distance for each experimental condition. The individual linear fits were very good, with *r*^2^ ranging from 0.79–1.0 across conditions (the mean *r*^2^ averaged across subjects and conditions was 0.96, *SD* = 0.06). A two-way repeated-measures ANOVA was conducted on the d′ threshold values with factors of displacement condition (target probed vs. context probed) and blank condition (no blank vs. blank). The main effect of displacement condition was significant, *F*_(1,5)_ = 22.5, *p* < 0.01, *MSE* = 1.08, as displacement thresholds were lower on target probed trials than on context probed trials. The main effect of blank condition was also significant, *F*_(1,5)_ = 13.8, *p* < 0.02, *MSE* = 0.21, as displacement thresholds were lower on no-blank trials than on blank trials. The interaction between displacement condition and blank condition was not significant, *F*_(1,5)_ = 1.44, *p* > 0.25, *MSE* = 0.26.

### Discussion

The results of Experiment 3 replicated those of Experiment 2 very closely. Displacement of the saccade target object was detected more accurately than displacement of other objects in the display, even though the saccade target object was probed on only 25% of trials. This provides further evidence that the saccade target object has privileged status in detecting instability across saccadic eye movements. Furthermore, presenting a post-saccadic blank interval before presenting the probe was found to hurt, rather than to help, the detection of displacements across the saccade. The proportion of no-displacement and displacement trials was equivalent in this experiment, so the increase in false alarms that we observed on displacement trials can not be attributed to there being more displacement than no-displacement trials. In sum, the results of the first two experiments seem not to have been due to biased sampling of the saccade target nor to unequal numbers of displacement and no-displacement trials.

## General Discussion

How the perception of a stable visual environment is achieved given unstable retinal input is a classic question in vision science. Recent theories of perceptual stability across saccades have focused on the role of the saccade target object; for example, the saccade target object theory of McConkie and Currie (1996) proposes that the perception of stability across saccades relies on a local evaluation process centered on the saccade target object rather than on remapping and evaluating the positions of all of the objects in a display. In support of such theories, several studies have found that if the object that the eyes are sent to maintains its position across the saccade, then stability is usually perceived, but if this object changes its position or experiences a change in its surface features then instability is usually perceived. In the present paper we examined whether perceptual stability relies only on the saccade target object or whether other objects in the visual field also contribute to the perception of a stable visual world across saccades. This question has received little study, as most studies of perceptual stability across saccades have only investigated perception of the saccade target, which is often the only item present in the visual field.

The results of our experiments show that the saccade target has high priority in determining the perception of stability across saccades. We found that the threshold for detecting displacement of the saccade target object was much lower than the threshold for detecting displacement of other objects in the visual field, and furthermore it was unaffected by the number of other items in the display. Perception and evaluation of the saccade target has two advantages: one is that visual attention precedes the eyes to the saccade location (e.g., Hoffman and Subramaniam, 1995; Kowler et al., 1995; Deubel et al., 1996; Irwin and Gordon, 1998), so the saccade target is preferentially encoded into memory; the second is that the fovea lands near the saccade target, so its position can be evaluated with high spatial resolution.

Our results suggest that other elements in the display are also evaluated across a saccade, however, but with lower resolution and limited by memory capacity. Objects farther from the fovea are seen with lower spatial resolution (e.g., Loschky et al., 2005; Strasburger et al., 2011), and thus there is more imprecision in people’s estimates of their locations (Rosenholtz et al., 2012), which makes it difficult to perceive whether a context item has moved or not during a saccade. In addition, however, we found that displacement detection of context items declined as the number of items in the visual field increased, indicating an attentional or memory capacity limitation on their influence in the perception of stability across saccades.

An unexpected result was our finding that the presentation of a post-saccadic blank hurt, rather than helped, displacement detection, largely by increasing false alarms. This was true both when the saccade target was probed and when a context item was probed. This contrasts with several other studies that have shown that adding a post-saccadic blank interval before presenting the probe improves the detection of the direction of stimulus displacements across saccades (e.g., Deubel et al., 1996, 1998; Demeyer et al., 2010; Tas et al., 2012). As we discussed earlier, our procedure differed in several respects from that used in these earlier studies. Perhaps most importantly, in the earlier studies the stimulus always moved and participants had to report in which direction it moved, whereas in our procedure sometimes the stimulus moved and sometimes it did not, and participants had to judge whether a movement had occurred. It is possible that the disruption in object correspondence caused by the blank interval led subjects in our experiments to perceive the post-saccadic probe as being *different* from its pre-saccadic precursor, but it is important to note that subjects had been clearly instructed in both written and verbal form to report whether they perceived *displacement*, rather than any type of *change* across the saccade. According to Deubel and colleagues, blanking the display should have led to more accurate judgments of spatial position because blanking the display allows the perceptual system to use high-fidelity information about eye position and precise information about the presaccadic target position to update spatial locations. Thus, if blanking the display does improve judgements of spatial position, as proposed by Deubel and colleagues, we would expect that subjects in our experiments would be more accurate at judging whether the stimulus moved or did not move during the saccade because precise information about its pre-saccadic location would be available. This did not happen. Given that all of the studies that have found beneficial effects of blanking on spatial discrimination across saccades have used a motion direction discrimination task, it seems possible that a post-saccadic blank may aid discrimination of motion direction across saccades rather than improving knowledge of spatial position *per se*. This requires additional research.

One potential criticism of our experiments is that they may be studies of spatial memory rather than studies of visual stability across saccades. This criticism misses the mark because all studies of visual stability are also studies of spatial memory. In order to perceive the world as stable (or unstable), the viewer must have spatial memory of where objects were before the saccade in order to determine whether they changed positions during the saccade. Every task that has ever been used to study perceptual stability across saccades is a spatial memory task, including displacement detection tasks like ours and (Bridgeman et al., 1975) and motion discrimination tasks like those used by Deubel and others–one can not judge which direction something moved unless they have memory for its starting location.

Given that perceptual stability relies on spatial memory, it is of interest to know what kind of memory is used in support of perceptual stability. Based on their finding that displacement detection performance for context items was as good when there were six items in a display as when there were only two, Irwin and Robinson (2014) proposed that informational persistence, a high-capacity, nonvisible, precategorical memory that codes form and location information in a precise format for approximately 300–500 ms after stimulus offset (Irwin and Yeomans, 1986; Di Lollo and Dixon, 1988) might underlie the perception of visual stability and visual displacement across saccades (see also Germeys et al., 2010). The present (more powerful) experiments are inconsistent with this hypothesis, however, because they show that there is a severe capacity limit on performance, with displacement detection being considerably worse when there are four or six items in a display than when there are two. This capacity limit suggests that perceptual stability may rely instead on visual working memory, a limited capacity memory that maintains schematic representations of visual stimuli across saccades rather than highly detailed visual images (e.g., Irwin, 1991; Carlson-Radvansky and Irwin, 1995; Irwin and Andrews, 1996; Irwin and Gordon, 1998; Hollingworth et al., 2008). Visual working memory has been shown to underlie gaze correction, which also relies on object correspondence across saccades (e.g., Hollingworth et al., 2008; Hollingworth and Luck, 2009).

In conclusion, the results of the present study show that the saccade target object has priority in determining the perception of stability across saccades, but other objects in the visual world contribute as well. Their influence is muted relative to the saccade target object because of acuity limitations, attentional limitations, and limitations on memory capacity.

## Conflict of Interest Statement

The authors declare that the research was conducted in the absence of any commercial or financial relationships that could be construed as a potential conflict of interest.
